# A Rare Case of Isolated Male Epispadias

**DOI:** 10.7759/cureus.46160

**Published:** 2023-09-28

**Authors:** Dawood Shehzad, Mustafa Shehzad, Abdul Mutaal

**Affiliations:** 1 Internal Medicine, Rawalpinidi Medical University, Rawalpindi, PAK; 2 Internal Medicine, University of South Dakota, Sioux Falls, USA; 3 Internal Medicine, Hackensack University Medical Center, Hackensack, USA; 4 Plastic and Reconstructive Surgery, Rawalpindi Medical University, Rawalpindi, PAK

**Keywords:** genito-urinary track, urinary incontinence, modified cantwell-ransley, bladder exstrophy-epispadias complex, isolated male epispadias

## Abstract

Epispadias is a congenital malformation marked by the failure of the urethral bulb to tubularize dorsally. This results in a wide-open urethral plate dorsally. Epispadias is frequently associated with bladder exstrophy-epispadias complex.

We report the case of a five-year-old patient who presented in the outpatient department with isolated epispadias without any other associated abnormality.

This report aims to document this rare case of isolated male epispadias with incontinence and the success of using the modified Cantwell-Ransley technique for its treatment.

## Introduction

Epispadias is a congenital malformation marked by the failure of the urethral bulb to tubularize dorsally. Contrary to hypospadias, where the meatus lies ventrally on the penile shaft, children with epispadias have a wide and open urethral plate that lies on the dorsum. Isolated male epispadias largely remain rare, with an estimated incidence of less than 1 per 100,000 live births [1]. More commonly, epispadias occurs associated with bladder exstrophy, resulting in the use of bladder exstrophy-epispadias complex (BEEC) to describe non-isolated epispadias cases [1]. Epispadias can be diagnosed clinically, but additional investigations, such as ultrasonography of the genitourinary system and cystourethrography, are needed to exclude associated congenital anomalies of the upper urinary tract [1]. Commonly associated abnormalities include pubic diastasis [1], vesicoureteric reflux [1], urolithiasis [2], and urethral duplication [3].

Surgical management of epispadias can be difficult, requiring significant expertise. Surgical aims include reconstructing the anatomy to provide optimal function and cosmetics for the genitals and urethra [1]. Currently, early mortality is no longer a significant concern due to advances in early diagnostic techniques, surgical techniques, and immediate postoperative care. Now, the focus lies on employing surgical techniques to maximize the quality of life post-repair. Reconstructive procedures and surgical repair of any cosmetic defect are paramount [4]. Two surgical techniques have gained widespread adoption for the repair of male epispadias. 

The modified Cantwell-Ransley repair technique involves mobilizing the urethral plate, moving it ventrally, and separating the corporal bodies while preserving the attachment of the distal plate to the glans. A reverse-MAGPI (meatal advancement and granuloplasty) procedure is performed on the distal urethra. The corporal bodies are medially rotated and approximated. The other technique, the complete penile disassembly technique, was described by Mitchell and Bagli in 1996 [5]. This technique capitalizes on the unique anatomy of epispadias. It allows for complete separation of the urethral plate from the corpora and hemiglans. This approach is advantageous as it facilitates better reconstruction and enables additional procedures like bladder neck repair. The choice between these techniques depends on the surgeon's expertise and the specific case.

This report aims to document this rare case of isolated male epispadias with incontinence and the success of using the modified Cantwell-Ransley technique for its treatment.

## Case presentation

**Figure 1:**
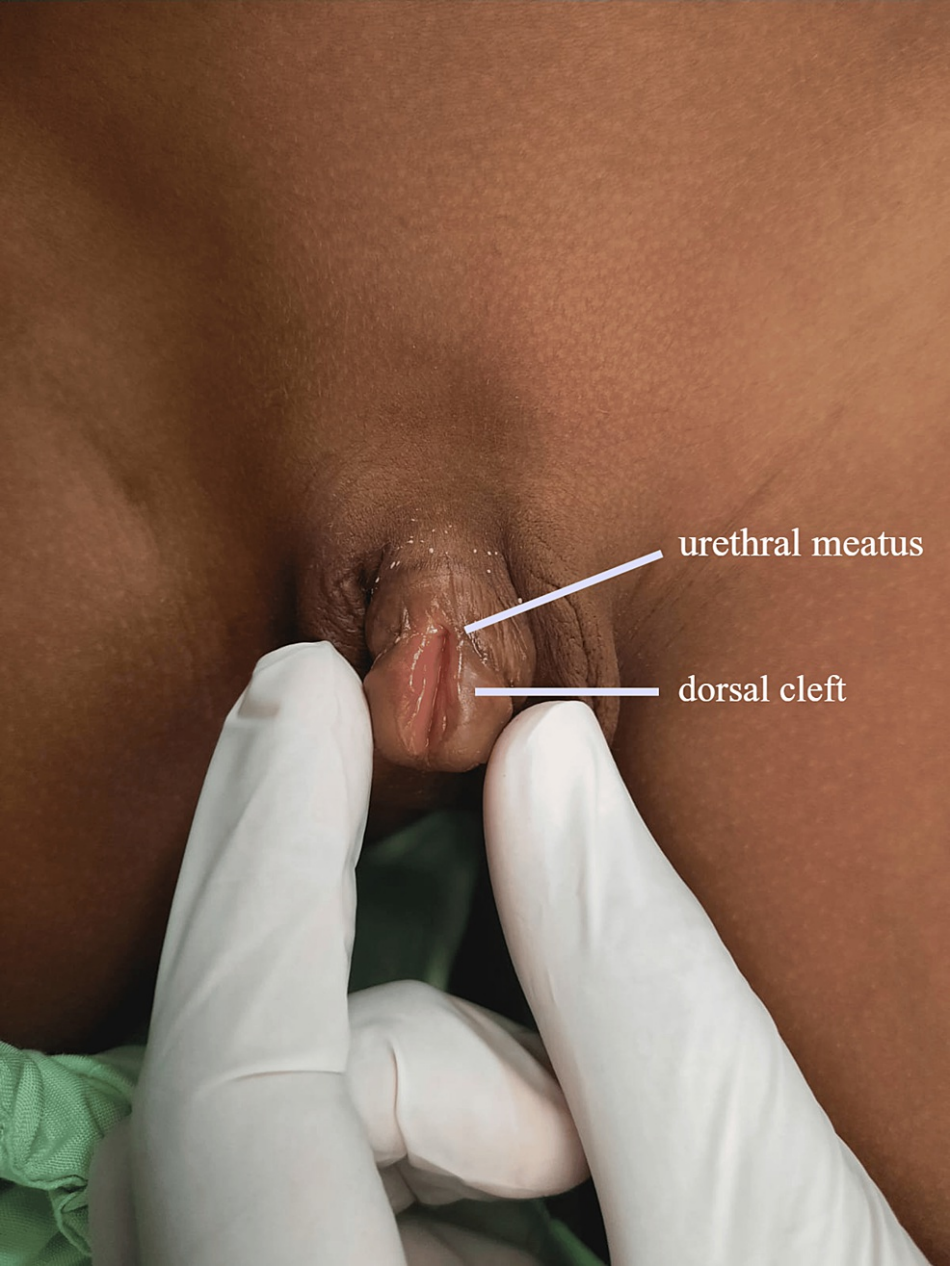
The preoperative anatomy indicating the position of the urethral meatus and dorsal cleft can be appreciated here.

A five-year-old patient presented in the plastic surgery outpatient department of Holy Family Hospital with complaints of an abnormally located external urethral orifice, micropenis, and urinary incontinence. There was no history of painful micturition, catheterization, family history of epispadias, or any other anomalies. On examination, a single external urethral meatus was found to be located at the junction of the glans and shaft of the penis. Moreover, there was a cleft on the dorsal surface of the glans. The size of the penis was less than normal (Figure 1). The scrotum, testis, abdominal, and rectal examinations were unremarkable.

**Figure 2:**
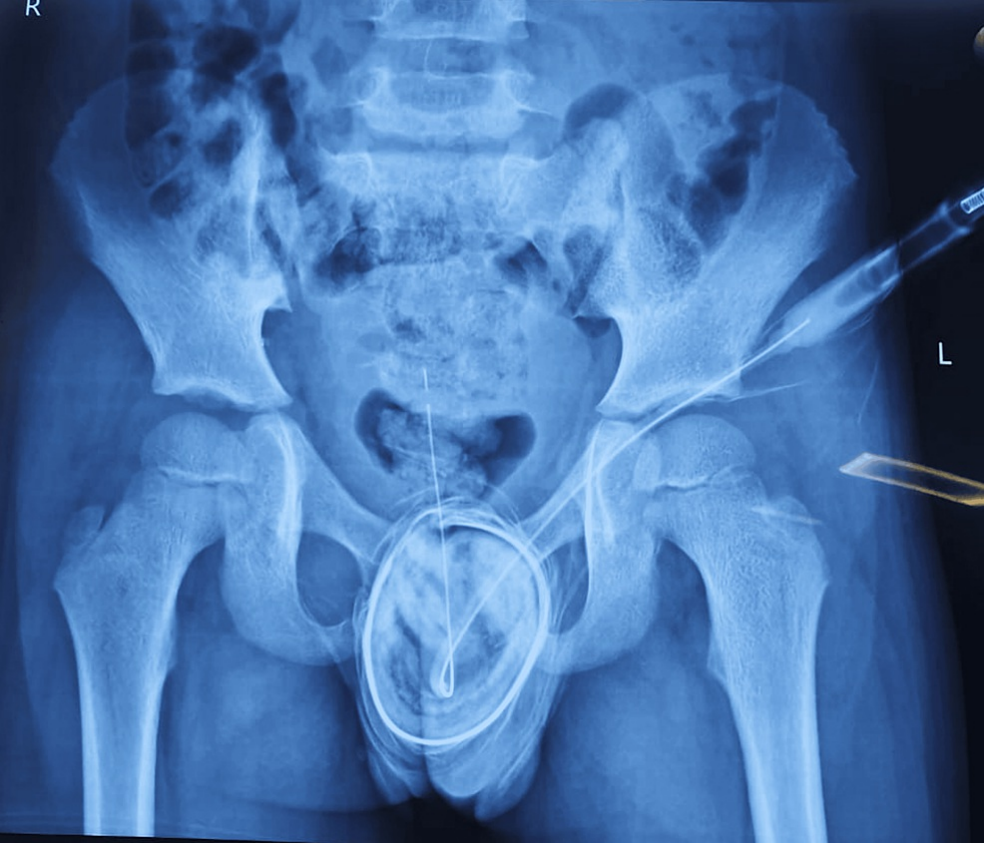
Pubic symphysis diastasis can be seen in the midline.

Ultrasonography (USG) of the abdomen and an X-ray of the pelvis were done. No genitourinary abnormality was detected on the USG abdomen except for the contracted gallbladder. X-ray of the pelvis has revealed pubic symphysis diastasis (Figure 2). A voiding cystourethrogram was not done due to the patient's financial constraints. 

The patient was prepared for surgery in the usual manner. 

Epispadias was repaired using the modified Cantwell-Ransley technique. It includes the following steps.

**Figure 3:**
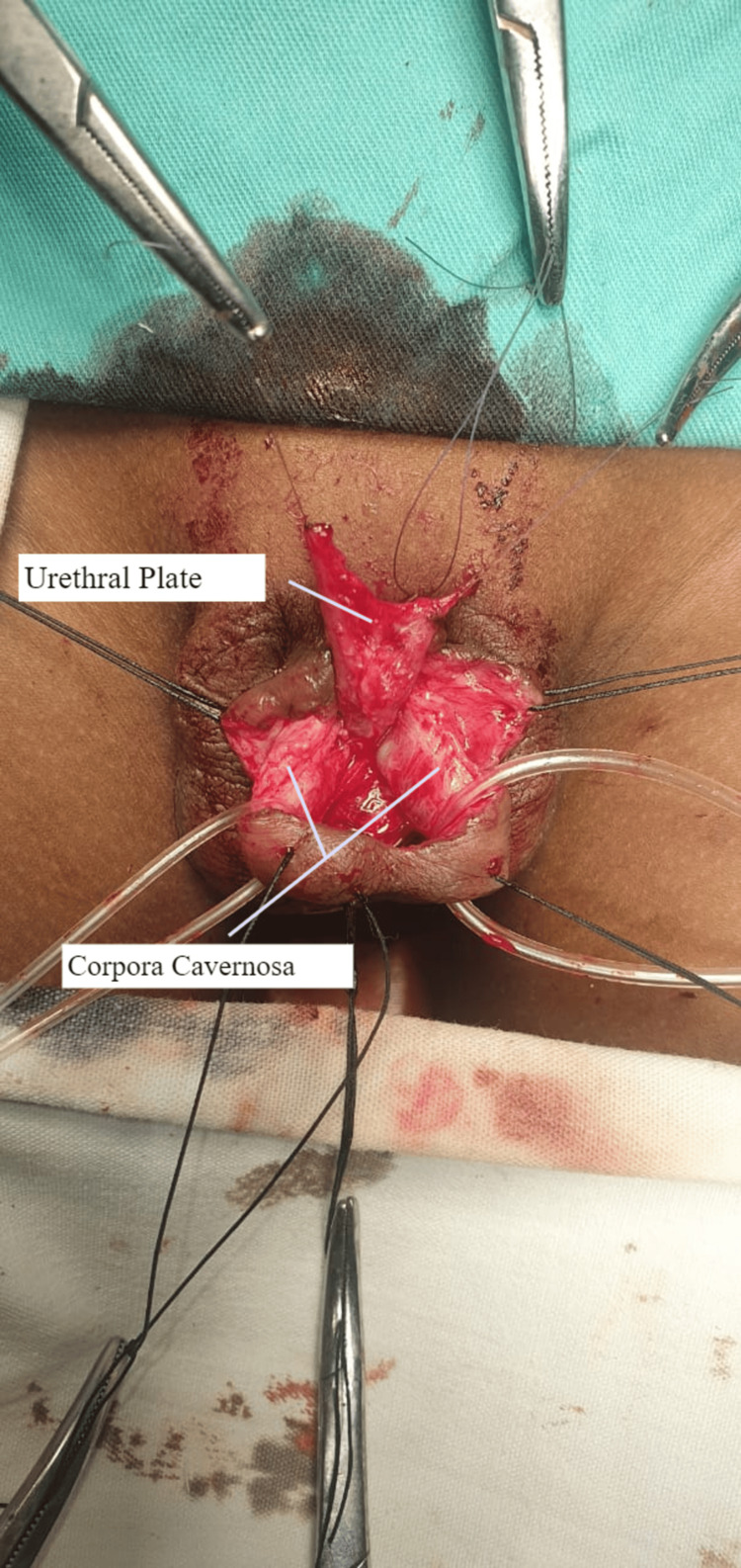
The corpora cavernosa, urethra, and the corpora spongiosum have been separated.

**Figure 4:**
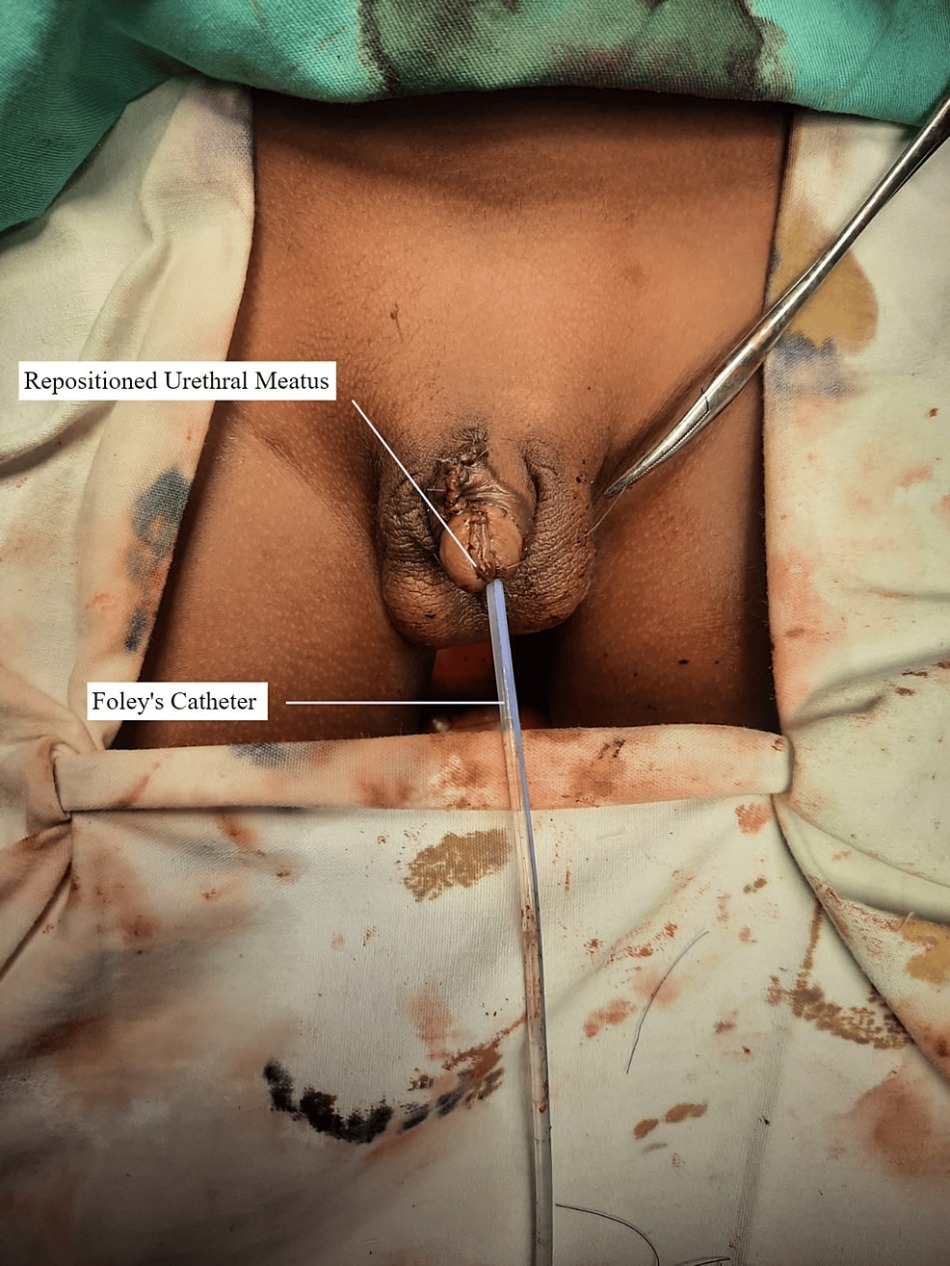
The urethral meatus has been repositioned.

1. Degloving of penile skin.

2. Separation of both corpora cavernosa, urethra, and the corpora spongiosum (Figure 3).

3. The urethral shape was tubularized. 

4. The urethra was moved ventrally, and lateral glans wings were created for a natural appearance. 

5. Reverse-MAGPI procedure on the distal urethra to refine the tip of the penis and position the urethral opening correctly. 

6. Finally, after medial rotation and approximation of the corporal bodies, the skin was closed, with repositioning of the meatus (Figure 4).

**Figure 5:**
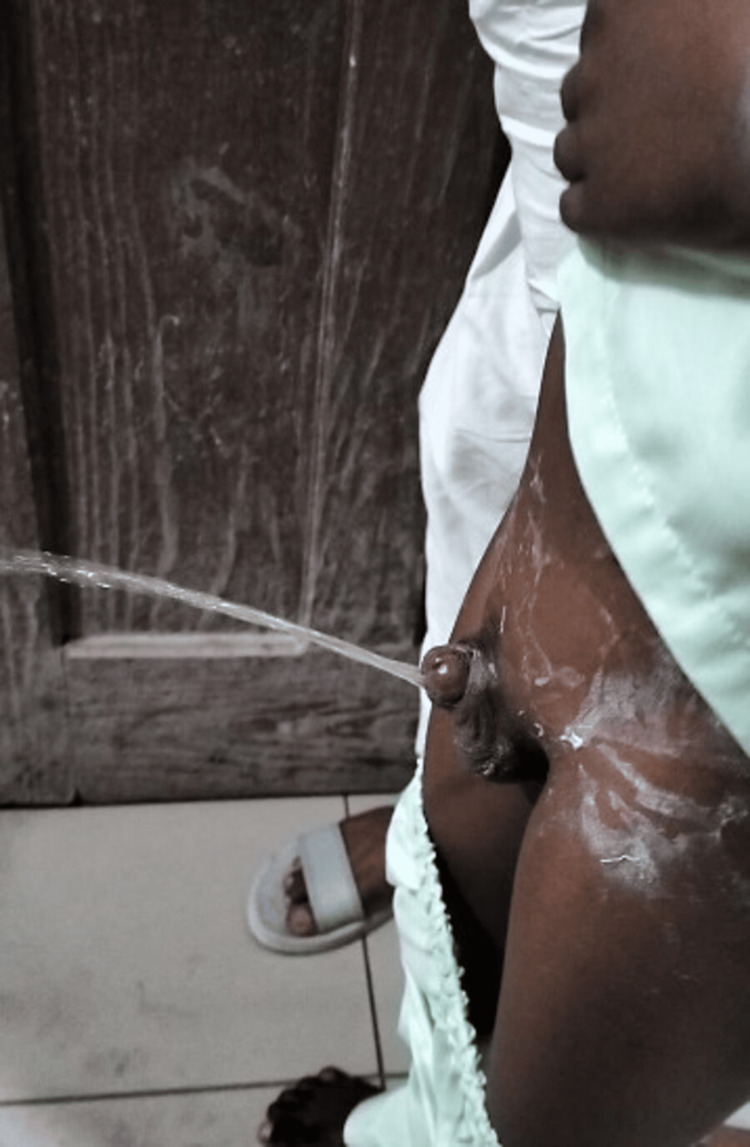
Urinary stream and function.

It was advised not to remove the Foley catheter for two weeks. Postoperatively, the patient complained of supra-pubic pain. The frequency and intensity of pain decreased with time. A follow-up visit was done after two weeks, and the Foley catheter was removed. Meatal position and urination were normal, and the patient did not complain of any incontinence. He had an excellent urinary stream and function (Figure 5).

## Discussion

Many techniques are reported in the literature for BEEC reconstruction [1], with variable success rates. The modified Cantwell-Ransley technique preserves the attachment of the distal urethral plate to the glans. This results in a more natural appearance of the glans and potentially better cosmetic outcomes.

However, the literature on isolated male epispadias is very scarce, and urinary continence in males as a primary outcome is not reported. This is rather unfortunate as continence is an essential issue in these children. Globally, different centers have mentioned variable continence rates depending on the type of epispadias, ranging from 50% to 90% [6].

Nocturnal continence rates rarely approach more than 50%. The main factors contributing to incontinence include the distorted anatomy of the bladder neck and the posterior urethra, along with histological abnormalities in the roof tissues [4]. Continence generally improves with pubertal onset; this indicates the importance of expectant management. It is proposed that contributions by the maturing prostatic tissues during puberty improve continence [7]. 

Pelvic X-ray and cystourethrogram should be done to look for any possible pubic symphysis diathesis and urogenital abnormalities. Postoperatively, pain is a common complication. Patients should be monitored periodically to look for any possible urinary incontinence and possible risk of procedure failure, which may require revision. Bladder spasms following repair are common, and anticholinergic medications should be started. These play a role in managing detrusor relaxation as well as provide a nominal degree of local anesthetic action. 

In this case of isolated male epispadias, the characteristic outward rotation of the innominate bones resulting in a wide pubic symphysis was appreciated via an X-ray pelvis. If very large, these are repaired with anterior and vertical osteotomies. 

Various surgical procedures have been reported in the literature to treat this anomaly. However, the repair is far from trivial and can be challenging even in the most experienced of surgical hands. Among the techniques described, the modified Cantwell-Ransley has become the procedure of choice due to excellent cosmetic, functional, and anatomical results in isolated epispadias [8]. 

Surgery aims to achieve a functionally and cosmetically acceptable penis [2]. It is important to keep in mind that superior rates of continence are noted when the reconstruction is performed by skilled surgeons [2]. Other factors that influence continence include the type of epispadias and associated penopubic defects. No studies to date have evaluated the effect of age at presentation and subsequent continence rates. General consensus favors early rather than late repair of these anomalies. Surgical repair should be attempted as soon as possible to avoid osteotomy. Of concern, however, is that adolescents with isolated epispadias may suffer from psychosocial and sexual issues.

Consultation should be obtained with psychiatry and urology/andrology during the transition of care into adulthood for comorbid conditions to be addressed properly.

## Conclusions

The purpose of this report is to document this rare case of isolated male epispadias with incontinence and the success of using the modified Cantwell-Ransley technique for its treatment. 

Most cases of epispadias are associated with other serious congenital anatomical anomalies, of which bladder exstrophy occurs most often. Finding isolated epispadias without associated anomalies is rare. However, in an isolated male epispadias case, it is essential to look for associated anomalies.
